# O009. Early pain relief from orthostatic headache and hearing changes, assessed with the visual analogue scale, in spontaneous intracranial hypotension after epidural blood patch in Trendelenburg position: 28 case reports

**DOI:** 10.1186/1129-2377-16-S1-A124

**Published:** 2015-09-28

**Authors:** Enrico Ferrante, Valentina Sangalli, Elena Olgiati, Fabio Rubino, Elio Agostoni

**Affiliations:** Neurosciences Department, Niguarda Cà Granda Hospital, Milan, Italy; Imperial College London, Department of Medicine, Division of Brain Sciences, London, UK and University of Milan-Bicocca, Department of Psychology, Milan, Italy; Anaesthesiology Department, Niguarda Cà Granda Hospital, Milan, Italy

## Background

Spontaneous intracranial hypotension (SIH) is characterized by orthostatic headache (OH), diffuse pachymeningeal enhancement on brain MRI and low CSF pressure. Hearing change (HC) is a frequent finding. Epidural blood patch (EBP) is now the most recommended available treatment. Our study aimed at investigating the EBP efficacy on OH and HC by asking patients to rate their OH and HC at different time intervals with a visual analogue scale (VAS).

## Materials and methods

Twenty-eight consecutive patients with SIH were treated with EBP in Trendelenburg position. Two Psychologists asked them to rate, on a VAS, the intensity of their OH and HC before, 24 hours after, and two months after treatment.

## Results

A significant improvement in OH and HC was found (p < .001) 24/48 hours after EBP. When followed-up, all patients showed complete relief from OH. Four patients out of 16 reported very mild HC.

## Discussion and conclusions

To the best of our knowledge, this is the first time a specific pain assessment with VAS was conducted before and after EBP, showing a fast improvement of OH and HC in a large group of SIH patients. Importantly, patients have been followed up for two months and 13-25 months after discharge, which confirmed the effect to be complete and long-lasting. In a future work, it may be worth monitoring patients’ changes over time with multiple follow-ups, also involving larger patients sample in a multicentric study.

Written informed consent to publication was obtained from the patient(s).
